# Диагностика и новые возможности лечения аденом гипофиза

**DOI:** 10.14341/probl13199

**Published:** 2023-05-11

**Authors:** А. Ю. Григорьев, В. Н. Азизян, О. В. Иващенко, Г. Ю. Старков

**Affiliations:** Национальный медицинский исследовательский центр эндокринологии; Национальный медицинский исследовательский центр эндокринологии; Национальный медицинский исследовательский центр эндокринологии; Московский государственный медико-стоматологический университет им. А.И. Евдокимова

**Keywords:** аденома гипофиза, эндоскопия, транссфеноидальный доступ, соматотропинома, кортикотропинома

## Abstract

В настоящий момент лечение аденом гипофиза неразрывно связано с транссфеноидальным нейрохирургическим вмешательством. Современные технологии, применяемые в хирургии при данной гипофизарной патологии, такие как эндоскопия с применением угловой оптики, а также использование специализированного инструментария, герметизирующего и гемостатического материалов повышают эффективность хирургического лечения аденом гипофиза и снижают частоту развития интраи послеоперационных осложнений. Развитие лучевых методов диагностики, таких как МРТ, позволяет с большей точностью выявить образование гипофиза, оценить его размеры, направление роста и степень инвазии окружающих тканей.

Авторы статьи подробно описали современную технику эндоскопического транссфеноидального удаления аденомы гипофиза. Каждый этап операции пошагово изложен с учетом различных анатомических особенностей и проиллюстрирован. Также в данной статье рассматриваются МР-характеристики аденом гипофиза: размер опухоли, направление ее роста, степень инвазии кавернозных синусов, компрессионный эффект на структуры хиазмально-селлярной области.

Применение современных методов диагностики и лечения в значительной степени повышает эффективность нейрохирургического вмешательства и позволяет снизить риски развития осложнений у пациентов.

## Abstract (en)

Currently, the treatment of pituitary adenomas is inextricably linked with transsphenoidal neurosurgical intervention. Modern technologies used in surgery for this pituitary pathology, such as endoscopy using angled optics, as well as the use of specialized instruments, sealing and hemostatic materials, increase the effectiveness of surgical treatment of pituitary adenomas and reduce the incidence of intraand postoperative complications. The development of radiation methods of diagnostics, such as MRI, makes it possible to more accurately identify the formation of the pituitary gland, assess its size, direction of growth, and the degree of invasion of surrounding tissues. The authors of the article described in detail the modern technique of transsphenoidal removal of pituitary adenoma using an endoscope. Each stage of the operation is described step by step, taking into account various anatomical features and illustrated. This article discusses the MRI characteristics of pituitary adenomas: size of the tumor, the direction of its growth, the degree of invasion of the cavernous sinuses, the compression effect on the structures of the chiasmal-sellar region. The use of treatment methods, knowledge of the features of MRI diagnostics described in this article greatly increase the effectiveness of the treatment of patients with pituitary adenomas and reduce the risk of complications after neurosurgical intervention in such patients.

## ВВЕДЕНИЕ

На сегодняшний день хирургия в значительной степени продвинулась в лечении аденом гипофиза благодаря применению эндоскопических технологий. Это произошло благодаря улучшенной визуализации зоны операции за счет введения эндоскопа высокого разрешения с широкоэкранной оптикой, позволяющей получать широкоугольное изображение интересуемой области. Применение эндоскопов с угловой оптикой (30, 45 и 70 градусов) позволило нейрохирургам увидеть структуры, находящиеся «за углом», что при определенном наклоне эндоскопа дает практически 180-градусный охват интересующей зоны (рис. 1, 2).

**Figure:**
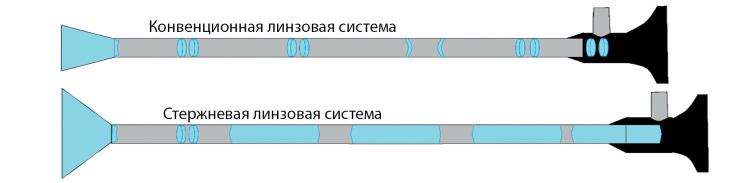
Рисунок 1. Эндоскопы с обычной и стержневой линзовой системой.

**Figure:**
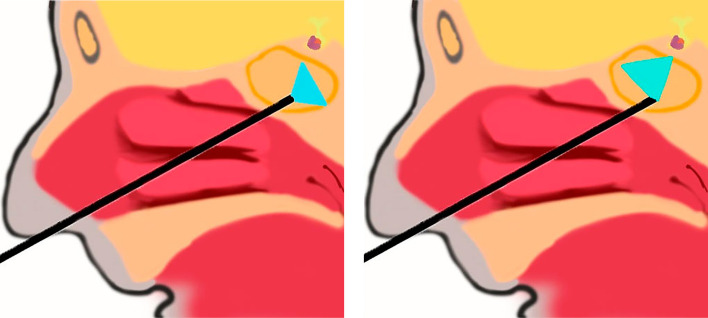
Рисунок 2. Угол видимости в прямой и угловой эндоскопы. Тем не менее эндоскоп сам по себе является лишь визуализационным прибором, а для проведения самого нейрохирургического вмешательства необходим комплект специализированного инструментария.

## ХИРУРГИЧЕСКАЯ ТЕХНИКА ЛЕЧЕНИЯ АДЕНОМ ГИПОФИЗА

Хирургическое лечение опухолей гипофиза включает три основных стадии, или этапа: назальный, сфеноидальный и селлярный. На каждой стадии хирургу необходимо пройти через те или иные структуры, применяя различные инструменты и оборудование. Если костные структуры выраженные и плотные, то хирургу приходится использовать высокоскоростной бор, которым можно высверлить отверстие нужного диаметра как в области входа в основную пазуху, так и в передней стенке турецкого седла для последующего осуществления хирургического удаления опухоли (рис. 3).

По завершении доступа к полости турецкого седла происходит рассечение твердой мозговой оболочки (ТМО) и оценка структуры опухоли гипофиза. Для этого применяются опухолевые кюретки, посредством которых происходит отделение опухоли от окружающих структур. При мягкой структуре аденомы происходит ее удаление опухолевыми кусачками и кюретками, при плотной структуре — дальнейшее вылущивание из окружающих тканей и затем удаление единым блоком или кускованием, если размер аденомы слишком большой.

«Темп удаления» опухолевой ткани зависит от ее консистенции. Кистозные опухоли удаляются быстро при помощи аспиратора. Большие студенистые или мягкие опухоли обычно «рождаются» под давлением после рассечения опухолевой капсулы и также легко удаляются аспиратором и тупыми кюретками и ложками. В случае плотных и тяжистых аденом, выраженной кровоточивости опухоли может потребоваться применение острых кюреток и опухолевых кусачек, а также вторая рука ассистента для одновременного захватывания опухоли, ее отсечения от стенок турецкого седла, а также одномоментной аспирации изливающейся крови. При установке держателя для эндоскопа у хирурга освобождаются обе руки, что значительно облегчает манипуляции в полости седла. Однако это не всегда удобно, особенно когда работа ведется с использованием угловой оптики в полости кавернозного синуса или супраселлярно. После удаления опухоли и капсулы ткань отправляется на морфологическое исследование.

Этапность удаления опухолевой ткани заключается в следующем: удаление начинается с нижней части опухоли, затем латеральных ее частей и в конце производится удаление супраселлярного компонента (при наличии такового) (рис. 4).

При удалении супраселлярной части опухоли диафрагма может быстро опуститься в полость седла и закрыть фрагменты опухоли по периферии в образовавшихся карманах, особенно в задней части диафрагмы. При такой ситуации рекомендуется регулярно контролировать низведение диафрагмы во время удаления опухолевой ткани и произвести ревизию образовавшихся карманов на наличие остатков опухоли, приподняв купол диафрагмы загнутой ложкой.

При удалении латеральных участков опухоли необходимо соблюдать крайнюю осторожность, поскольку медиальная стенка кавернозного синуса очень тонка, и ее легко можно повредить.

Как было уже сказано выше, заключительным этапом удаляется супраселлярная часть опухоли. При этом используется тупая кюретка, поскольку арахноидальная оболочка зачастую бывает очень тонка и может развиться ликворея даже при очень деликатных манипуляциях. Если диафрагма опускается неравномерно, карман с оставшейся там опухолью может располагаться позади опустившегося купола. В этой ситуации нужно деликатно приподнять диафрагму для визуализации оставшейся опухоли. После того, как опухоль удалена, 4 мм угловыми эндоскопами 30-, 45- и 70-градусными обследуются латеральные и супраселлярная области для выявления остаточной ткани, наличия ликвореи или дефектов твердой мозговой оболочки (ТМО) (рис. 2).

При развитии интраоперационной ликвореи желательно идентифицировать интраселлярный дефект, через который истекает спинномозговая жидкость. Необходимо соблюдать чрезвычайную осторожность, чтобы не сделать его больше и не ухудшить ситуацию и, по возможности, прекратить хирургические манипуляции вокруг него. В зависимости от выраженности дефекта его можно заклеить кусочком клеящейся пластины Тахокомб, аутожиром, мышцей или фасцией (например, в тех случаях, когда дефект образовался в результате слишком высоко сделанного вертикального разреза) или уложить в полость седла аутоткань и залить фибриновым клеем. После этого необходимо провести пластику дна турецкого седла, по возможности сшив края ТМО узловыми швами, или использовать специальные клипсы, позволяющие избежать кропотливой процедуры завязывания узлов в глубине носовой полости и сверху наложить заплату из ауто- (фасция, кусочек мышечной или жировой ткани) и/или синтетических материалов, скрепив все фибриновым клеем (рис. 5).

После окончания хирургических манипуляций необходимо обеспечить тщательный гемостаз. Небольшое просачивание или кровотечение из ветви клиновидно-небной артерии останавливается би- или монополярной коагуляцией. Для лучшего заживления слизистой оболочки полости носа необходимо проконтролировать, чтобы рострум клиновидной кости был адекватно закрыт остатками слизистой. Сверху можно уложить специальную назальную губку типа Мероцель, Гельфом, которую орошают физраствором и удаляют спустя 24–48 ч, а небольшие размеры губки позволяют пациенту дышать, не испытывая особого дискомфорта.

Первые сутки пациент должен находиться в палате интенсивной терапии для стабилизации состояния, оценки гемодинамики, выявления и купирования ранних послеоперационных осложнений. В первые дни очень важно обращать внимание на возможные проявления надпочечниковой недостаточности, а также развитие несахарного диабета для своевременного купирования их. Для этого ориентируются как на клиническую картину, так и на данные гормонального исследования.

**Figure:**
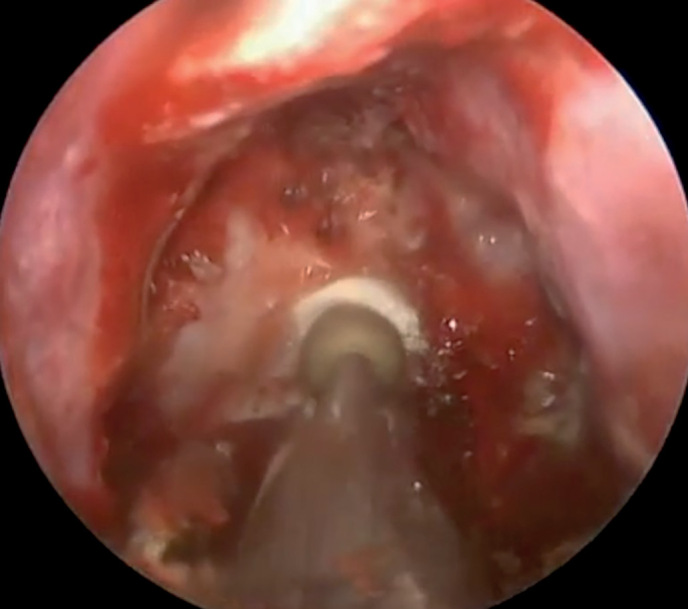
Рисунок 3. Фрагмент операции во время осуществления доступа через костные структуры основной пазухи.

**Figure:**
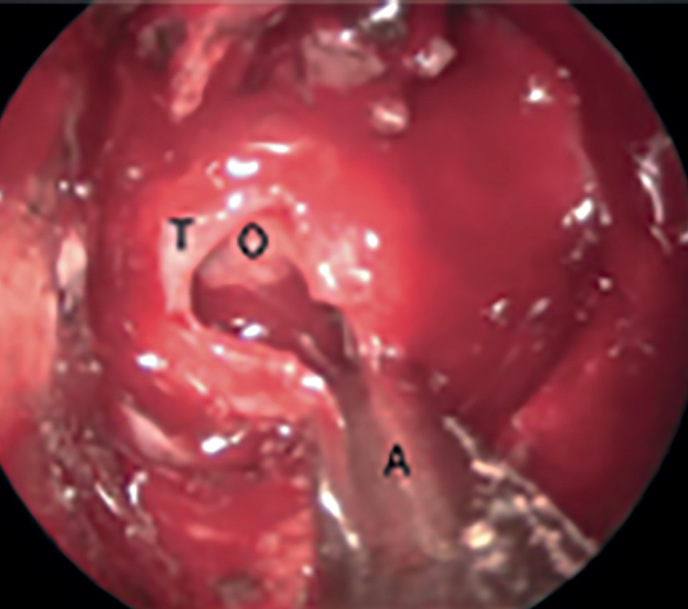
Рисунок 4. Процесс удаления опухоли из полости турецкого седла при помощи аспиратора (Т — твердая мозговая оболочка, О — опухоль, А  — аспиратор).

**Figure:**
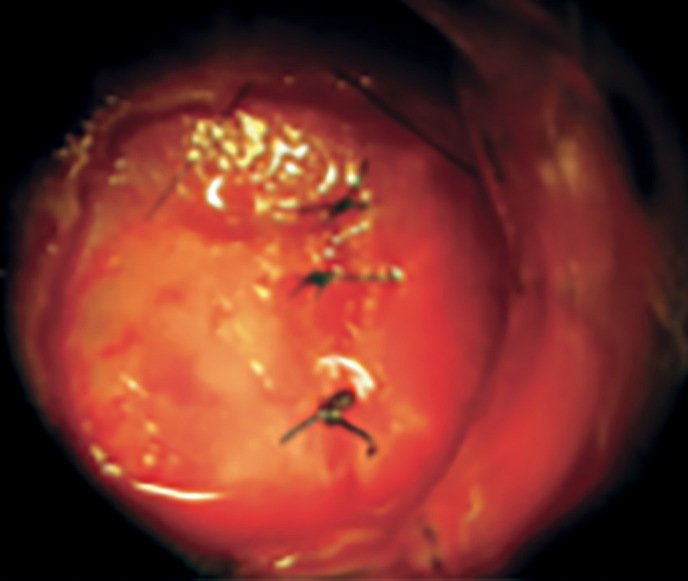
Рисунок 5. Ушитая ТМО после удаления опухоли гипофиза.

## МР-ХАРАКТЕРИСТИКИ АДЕНОМ ГИПОФИЗА

Результаты лечения аденом гипофиза зависят не только от правильной и своевременной диагностики, но и характера роста опухоли. Одна из ведущих ролей в диагностике аденом гипофизов принадлежит лучевым методам исследования — компьютерной (КТ) и магнитно-резонансной томографии (МРТ) (рис. 6–8).

Методом выбора в выявлении аденом гипофиза, в особенности микроаденом, благодаря своей высокой разрешающей способности является МРТ. Однако и КТ оказывает существенную диагностическую помощь в случаях невозможности проведения МРТ (наличие кардиостимулятора, эндопротеза), а также способствует лучшему выявлению костных изменений в полости носа, клиновидной пазухе, селлярной и латероселлярной областях.

Обычно в норме у взрослых высота гипофиза у мужчин составляет не более 8 мм, а у женщин не более 10 мм. Аденогипофиз на КТ гомогенной плотности, на МРТ имеет изоинтенсивный сигнал, и в обоих случаях характерно равномерное накопление контрастного вещества. В отличие от него для нейрогипофиза характерен гиперинтенсивный сигнал на МРТ в Т1-взвешенном режиме.

На МРТ микроаденомы в большинстве случаев имеют гипоинтенсивный сигнал в Т1-взвешенном режиме, реже изоинтенсивный. В случае микроаденом гиперинтенсивный сигнал в Т1-режиме может свидетельствовать о кровоизлиянии в опухоль, что более характерно при пролактиномах. В Т2-взвешенном режиме интенсивность сигнала различна и может быть от гиперинтенсивного до гипоинтенсивного. При введении контрастного вещества характерно значительно менее интенсивное накопление его опухолью по сравнению с нормальной тканью гипофиза.

Для макроаденом (размер опухоли >1 см) часто характерен экстраселлярный рост. Опухоль может иметь супраселлярный, инфраселлярный, ретроселлярный, антеселлярный, латероселлярный рост и их сочетания. МРТ позволяет определить взаимоотношение опухоли с окружающими структурами: зрительными нервами, хиазмой, кавернозными синусами, ВСА, дном третьего желудочка, что важно при планировании хирургических доступов. Большинство макроаденом гипофиза имеют гипоинтенсивный сигнал в Т1-режиме и гиперинтенсивный сигнал в Т2-взвешенном режиме (рис. 9, 10). Кроме того, они могут иметь как гомогенную, так и гетерогенную структуру, а также содержать кистозный компонент. Введение контрастного вещества помогает дифференцировать ткань опухоли, для которой характерно менее интенсивное накопление контрастного вещества, от нормальной ткани гипофиза. В случаях больших аденом ткань гипофиза бывает настолько сдавлена, что может не определяться на МРТ.

При макроаденомах с экстраселлярным ростом часто наблюдается распространение ее в кавернозные синусы. Однако четко выявить наличие роста в кавернозный синус весьма затруднительно. Связано это с тем, что медиальная стенка синуса довольно тонкая и не всегда возможно ее визуализировать. Наличие же роста опухоли между внутренней сонной артерии (ВСА) и латеральной стенкой синуса служит надежным признаком ее инвазии.

**Figure:**
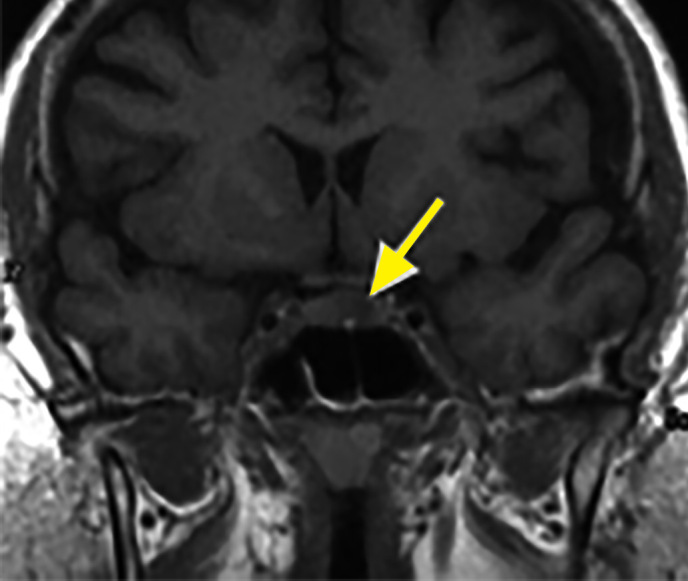
Рисунок 6. Эндоселлярная кортикотропинома. На фронтальных МР-томограммах в Т1-взвешенном режиме выявляется гипоинтенсивная опухоль (стрелка).

**Figure:**
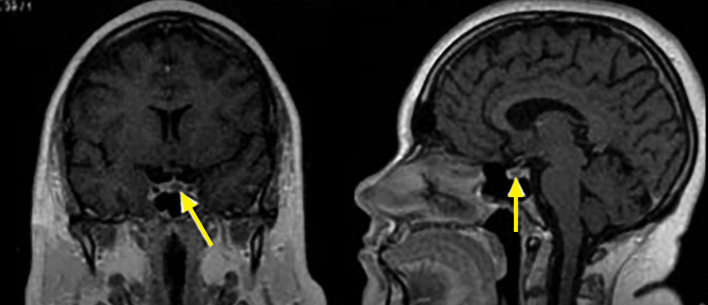
Рисунок 7. Эндоселлярная кортикотропинома. На фронтальных (А) и сагиттальных (Б) МР-томограммах в Т1-взвешенном режиме при введении контрастного вещества отмечается сниженное накопление контрастного вещества, в отличие от нормальной ткани гипофиза.

**Figure:**
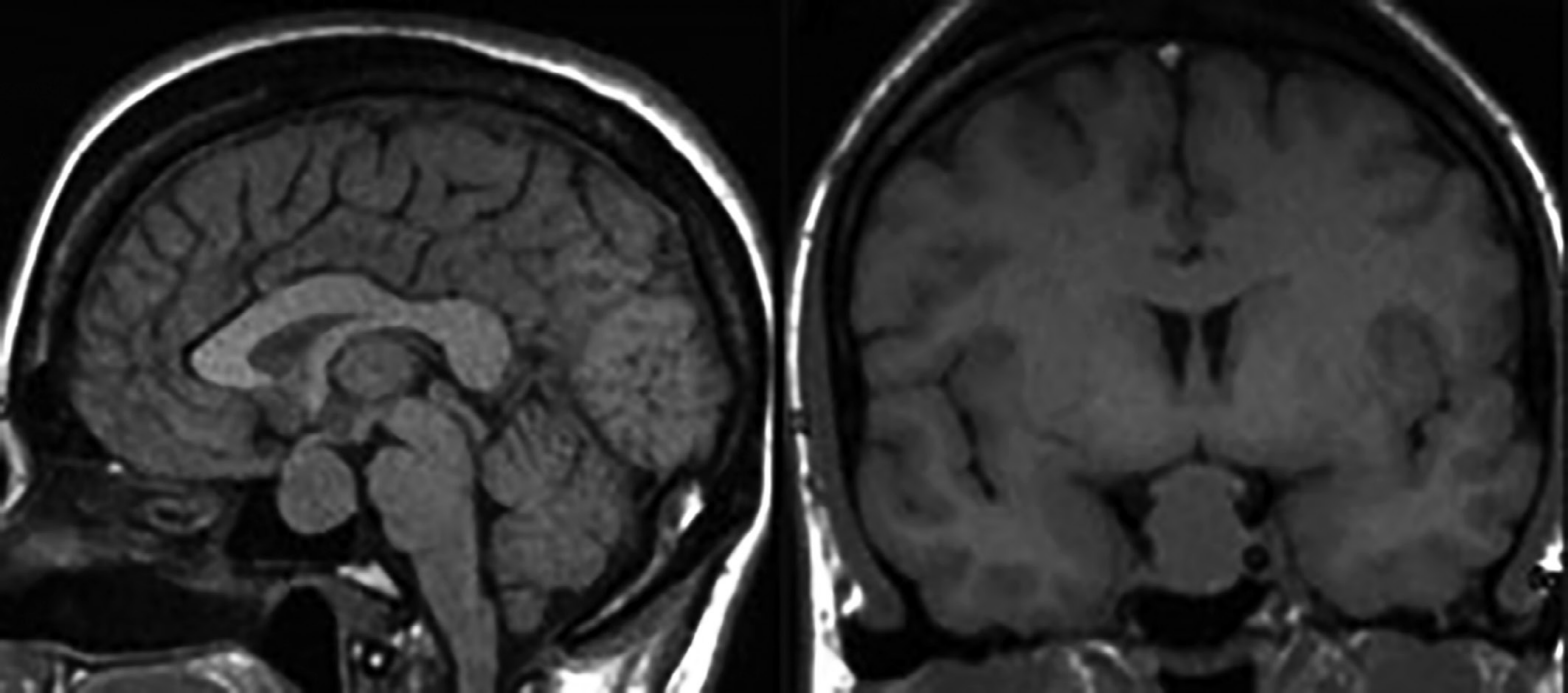
Рисунок 8. Эндо-супраселлярная соматотропинома. На сагиттальных (А) и фронтальных (Б) МР-томограммах в Т1-взвешенном режиме выявляется гипоинтенсивная опухоль.

**Figure:**
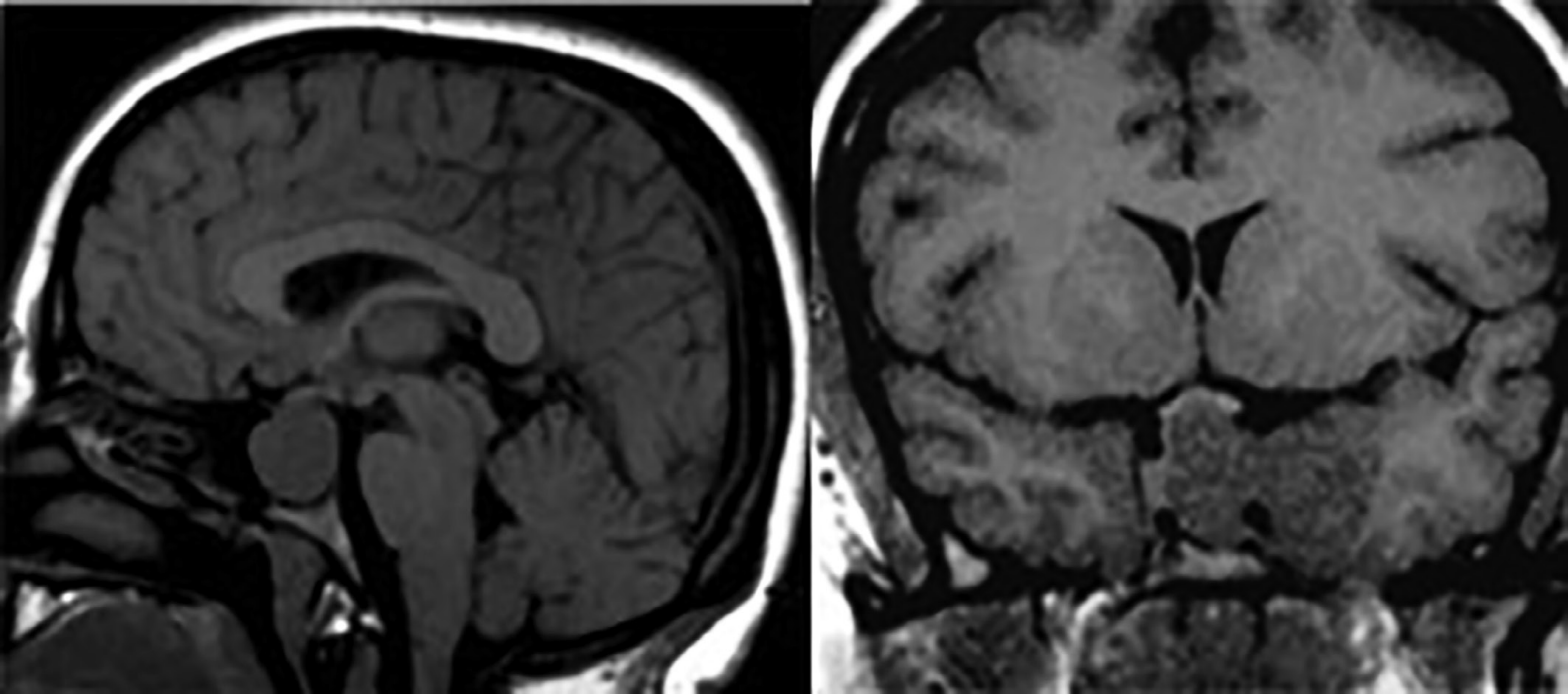
Рисунок 9. Эндо-супра-инфра-латероселлярная соматотропинома. На сагиттальных (А) и фронтальных (Б) МР-томограммах в Т1-взвешенном режиме выявляется гипоинтенсивная опухоль с ростом в пазуху клиновидной кости, супраселлярно с компрессией хиазмы зрительных нервов, латероселлярно — в левый кавернозный синус с обрастанием сифона ВСА и сдавлением медиальных отделов височной доли.

**Figure:**
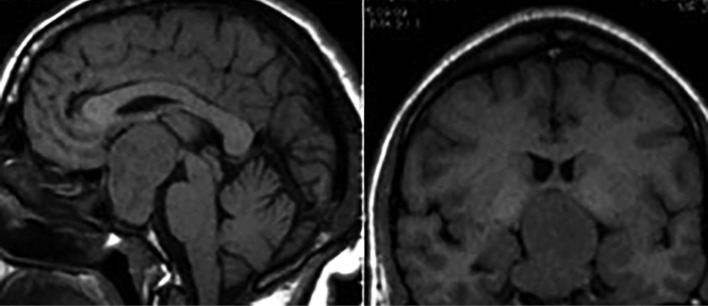
Рисунок 10. Эндо-супра-инфра-латероселлярная соматотропинома. На сагиттальных (А) и фронтальных (Б) МР-томограммах в Т1-взвешенном режиме выявляется гипоинтенсивная опухоль с ростом в пазуху клиновидной кости, супраселлярно с компрессией дна 3-го желудочка, латероселлярно — в правый кавернозный синус.

## ЗАКЛЮЧЕНИЕ

В завершение хотелось бы сказать, что, как правило, пациенты легко переносят эндоскопические операции, послеоперационные осложнения развиваются редко и в легкой форме. Безусловно, это зависит от размеров опухоли, выраженности экстраселлярного роста и степени дооперационных обменных нарушений. Естественно, возможно развитие более грозных осложнений, как эндокринологических, так и хирургических, включая послеоперационную ликворею, кровотечение, образование гематомы в ложе удаленной опухоли и даже летальный исход.

## ДОПОЛНИТЕЛЬНАЯ ИНФОРМАЦИЯ

Источники финансирования. Работа выполнена по инициативе авторов без привлечения финансирования.

Конфликт интересов. Авторы декларируют отсутствие явных и потенциальных конфликтов интересов, связанных с содержанием настоящей статьи.

Участие авторов. Григорьев А.Ю. — вклад по критерию 1, по критерию 2; Азизян В.Н. — вклад по критерию 1, по критерию 2; Иващенко О.В. — вклад по критерию 1, по критерию 2; Старков Г.Ю. — вклад по критерию 1, по критерию 2. Все авторы одобрили финальную версию статьи перед публикацией, выразили согласие нести ответственность за все аспекты работы, подразумевающую надлежащее изучение и решение вопросов, связанных с точностью или добросовестностью любой части работы
